# Impact of Body Mass Index on Muscle Strength, Thicknesses, and Fiber Composition in Young Women

**DOI:** 10.3390/ijerph19169789

**Published:** 2022-08-09

**Authors:** Eun-Sook Sung, Ahreum Han, Timo Hinrichs, Matthias Vorgerd, Petra Platen

**Affiliations:** 1 Department of Physical Education, Korea University, Seoul 02841, Korea; 2 Department of Sports Medicine and Sports Nutrition, Faculty of Sport Science, Ruhr-University Bochum, 44801 Bochum, Germany; 3 Division of Sports and Exercise Medicine, Department of Sport, Exercise and Health, University of Basel, 4052 Basel, Switzerland; 4 Department of Neurology, Ruhr-University Bochum, Kliniken Bergmannsheil, 44801 Bochum, Germany

**Keywords:** body mass index, menstrual cycle, muscle diameter, muscle strength, muscle fiber type

## Abstract

High body mass index (BMI) may influence muscle strength, muscle thickness (Mtk), and fiber composition. We evaluated these parameters in 31 and 27 women grouped in non-oral contraceptive (non-OC) groups and OC groups, respectively, and further divided them into groups based on BMI: BMI_low_, BMI_norm_, and BMI_high_. Maximum isometric force (F_max_), Mtk, and the relative percentage of muscle fiber composition (%) were examined in both groups. F_max_ and Mtk values were significantly greater in the BMI_high_ than the BMI_low_ within the OC group. However, there was no significant difference in the non-OC group. BMI_low_ and BMI_norm_ groups showed a difference in the distribution of muscle fiber types 1 and 2 with almost the same proportions in both non-OC and OC groups. However, the BMI_high_ group showed a difference in the distribution of muscle fiber types 1 and 2, with type 1 about 18.76% higher in the non-OC group. Contrastively, type 2 was about 34.35% higher in the OC group. In this study, we found that there was a significant difference in F_max_ and Mtk according to the BMI level in the OC group, but no significant difference was found in the non-OC group. Moreover, the distribution of type 2 muscle fibers tended to be higher in the OC group of BMI_high_, although the sample size was small. Therefore, although no significant difference of F_max_ and Mtk was found according to BMI level in the non-OC group in this study, the increase in BMI level appeared to be more associative of muscle strength in the OC group. Based on the present results, future studies are needed that consider the BMI level as well as the presence or absence of OC in future research about women’s muscle strength.

## 1. Introduction

The menstrual cycle refers to the time interval between the first day of the index and the following period. It is a dynamic and complex process, involving steroid hormones and fluctuations of endogenous estradiol and progesterone levels. The events of the cycle depend on the cell structure of the ovaries, which respond to hormonal signaling by the anterior pituitary gland [1]; the production of hormones by the ovaries is predictable and cyclical within an average of 23–38 days [2,3]. Reis et al. [4] conducted the first interventional study investigating menstrual cycle phase-based strength. They reported an association between the increased strength and endurance of the quadriceps muscle group and elevated estrogen levels (i.e., in late follicular and early luteal phases). Following their results, many subsequent studies have been conducted in the field.

Until now, previous studies reported that *endogenous female sex hormones* contribute to strength performance [5,6,7], but the results were inconsistent [8,9,10]. According to a recent review [11], it was reported that the menstrual cycle must be taken into consideration in women who do not use oral contraceptives (OC), and individual exercise programs should be mandated for the early follicular phase and all other menstrual cycle phases.

On the contrary, in women consuming exogenous hormones, endogenous hormone fluctuations are unlikely, and endogenous hormone release generally follows a predictable pattern; exogenous hormone supplementation involves taking hormones for 21 days (consumption phase), followed by a 7-day break (withdrawal phase). Consequently, endogenous estrogen and progesterone are suppressed, and according to the intake of constant amounts of exogenous hormone, blood concentrations of estrogen and progesterone remain nearly constant during the 21-day consumption phase [10]. Some evidence suggests that the levels of both endogenous and exogenous female sex steroids fluctuate, affecting exercise performance, strength gain, and anaerobic power [5,12,13,14]. Based on each woman’s response to OC use, along with other factors such as the primary objective for using OCs, a personalized therapeutic approach should be used to achieve better outcomes [15]. Nonetheless, these inconsistent results in menstrual cycle research on exercise performance may be related to methodological problems that mask possible changes during the cycle [16].

Several studies proposed that body weight and weight gain values are critical for the onset of menarche [17,18], and increased subcutaneous fat percentage and BMI levels were associated with menstrual pattern [19]. Thus, BMI strongly correlates with age at menarche and a regular menstrual cycle. The levels of luteinizing [20,21] and follicular stimulating hormones [20,21,22] and progesterone [20,21,23] are significantly lower in the ovulatory phase in obese women than in normal-weight women. Although it is unlikely that gonadotrophins are sequestered in the body fat, this may be the case for lipophilic sex steroids (i.e., progesterone) [24,25,26]; such sequestrated steroid hormones could create a sustained negative feedback loop that involves the hypothalamus and pituitary gland, suggesting that obesity may affect sex steroid hormone regulation in women. In addition, BMI is associated with the strength and cross-sectional area (CSA) of skeletal muscle. Indeed, overweight and obese individuals tend to have greater maximum voluntary contraction and CSA values than normal-weight individuals [27]. These findings are supported by Zoico et al. [28] and Rolland et al. [29]; both studies reported that muscular force and CSA values were 17.0% higher in obese than normal-weight women. However, little is known about the difference of the BMI level on muscle strength and thickness in women, including those who take OC and those who do not.

Therefore, the purpose of this study is to investigate the different muscle strength and muscle thickness in the non-OC and OC group, according to the BMI level. Furthermore, distribution of *muscle fibers* is investigated according to the BMI level in the non-OC and OC group.

## 2. Materials and Methods

**Figure 1:**
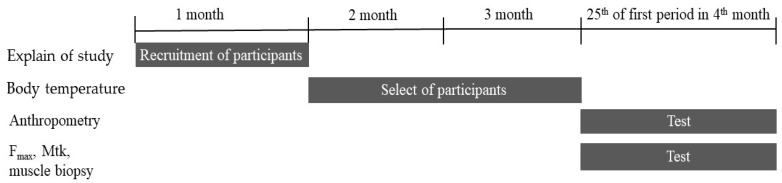
Flow diagram for the selection of study participants and experimental procedure.

This study involved 58 female participants who were students from Ruhr-university Bochum in Germany. The participants were randomly selected and the exclusion criterion was any health disorder. The participants were either untrained or moderately trained students that performed less than 2 h of regular physical exercise per week. A total of 31 eumenorrheic healthy women were included in the non-OC group (10, 10, and 11 women were included in the BMI_low_ < 18.5, 18.5 ≤ BMI_norm_ < 25, and BMI_high_ ≥ 25 groups, respectively). In addition, 27 women were included in the OC group (9 per BMI group). Women in the non-OC group had not been taking oral contraceptives or any other hormonal treatments for at least one year prior to participation in this study, and these women did not have a history of any endocrine disorders. Additionally, all women had regular menstrual cycles. Women in the OC groups had been taking monophasic combined OC for at least one year prior to participation in this study and did not have a history of any endocrine disorders (Table 1). The anthropometric data included mean age, height, weight, and BMI values. The participants were informed about the purpose, procedures, and risks associated with the present study before enrolment. All participants provided written informed consent (Figure 1). The study protocol was approved by the Ethics Committee of the Ruhr-University Bochum, Germany (IIA1-070118/07).

### 2.1. Menstrual Cycle Monitoring

Women’s body temperature naturally changes during the menstrual cycle. It is lower in the first part of the period and increases during ovulation. The occurrence of ovulation was defined when an increase in basal body temperature of at least 0.3 °C was measured [30,31,32]. This fluctuation of basal body temperature was used to identify people who have a normal menstrual cycle. Moreover, the phases of the menstrual cycle including ovulation are noted in order to individually determine the exact testing schedule (25th day of first period). Between 8:00 a.m. and 8:30 a.m., before rising from bed, the participants were requested to measure their basal body temperature daily using an oral electronic thermometer [33]. When no increase in basal body temperature, i.e., ovulation, was detected during both menstrual cycles, the subject was excluded from the study because she was judged not to have a normal cycle of menstruation.

### 2.2. Maximum Isometric Force (F_max_) Measurement

The maximum isometric force (F_max_) was determined on a leg press machine (Medizinische Sequenzgeräte, Compass, Germany) using a combined force and load cell (GSV-2ASD, ME-Messsysteme GmbH, Hennigsdorf, Germany). The temperature in the labor room was 20 °C with 50% humidity. F_max_ was measured individually for each participant on the 25th day following the first day of their menstrual cycle. Before testing, the participants underwent a 10-min warm-up on a low-resistance bicycle ergometer. They were familiarized with the test and the testing position (knee angle 90°, ankle angle 90°) during the leg press task [34]. Each measurement was repeated three times with 30 s of rest between trials. The highest value was selected among the three measurements for data analysis. A reliability analysis was performed for the isometric measurement; the intraclass correlation coefficient (ICC) was 0.998, which indicates that the system had a high internal consistency and, thus, a high reliability. 

### 2.3. Muscle Thicknesses (Mtk) Measurements

Mtk of the rectus femoris and vastus lateralis of each leg was measured using real time ultrasound imaging, which has been shown to be a reliable method [35]. We used a Vivid I CE 0344 ultrasonography device (GE Medical System, Solingen, Germany) with a parallel scanner (8 L-RS, 4.0–13.3 MHz), which provides a 10-cm depth of sound wave penetration, enabling the analysis of deep lying muscles. Mtk was measured using long-lasting static muscular tension that participants were asked to maintain for at least 30 min before the measurement [36]. Participants were in the supine position, and the ultrasound images were obtained at a point exactly half-way between the anterior superior iliac spine and the upper margin of the patella. The position of the transducer was recorded for each muscle so that it could be precisely reproduced during subsequent measurements. The mean value of the three measurements per muscle was taken for both legs, and the sum of the two Mtks was calculated for both sides of the body. A reliability analysis was performed for Mtk determination. The obtained ICC was 0.997, indicating a high reliability of the ultrasound imaging of Mtk used in this study.

### 2.4. Analysis of Muscle Fiber Composition

**Figure 2:**
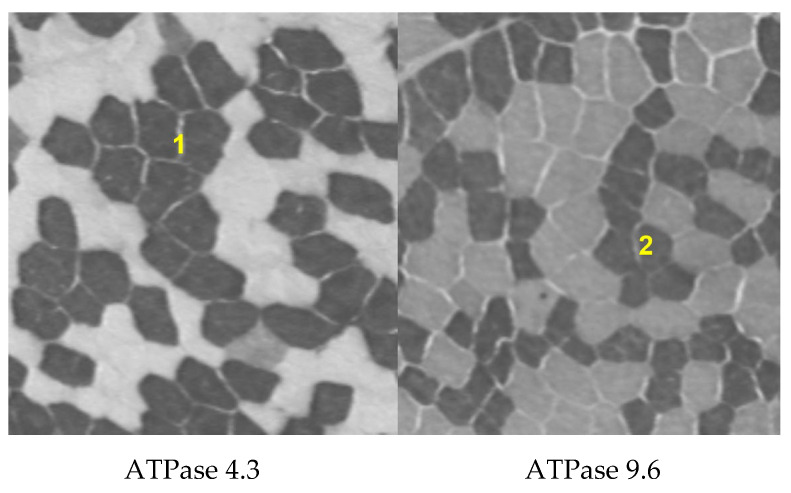
Histochemical analysis used to determine the proportion of muscle fiber types 1 and 2 using adenosine-triphosphatase staining.

Although the composition of different muscle fiber types is not uniform throughout the body, the composition of the vastus lateralis is considered to be a good indicator of the proportions of fiber types present in other major muscles involved in propulsive or working activities [37,38]. A total of 10 and 5 participants from the non-OC and OC groups, respectively, volunteered to participate in muscle needle biopsies. Under local anesthesia, percutaneous muscle biopsy samples (70–300 mg) were obtained from the vastus lateralis muscle using a previously described technique [39]. Directly after sampling, the tissue was removed from the needle, mounted as a cross-sectional slide in a Tissue-TEK^®^ embedded medium, frozen in isopentane, and placed into an aluminum container to be cooled further with liquid nitrogen before being stored at −80 °C for subsequent analysis [40]. We performed a histochemical analysis using adenosine-triphosphatase (ATPase) staining with alkaline pre-incubation at a pH of 4.3 and 9.6 to determine the proportion of muscle fiber types 1 and 2, respectively [41] (Figure 2). All fibers from a single sample were counted twice; the average of the values obtained was used in the statistical analysis [42].

### 2.5. Statistical Analysis

A statistical analysis was performed using IBM SPSS Statistics for Windows/Macintosh, ver. 22 (IBM Corp., Armonk, NY, USA). A one-way ANOVA was used to compare the differences in variance among the three BMI groups (BMI_low_, BMI_norm_, and BMI_high_) within the non-OC and OC groups. The Bonferroni correction was used as a post-hoc test to verify the statistical significance of the findings. *p*-values of < 0.05 were considered to be statistically significant.

## 3. Results

In the OC, the BMI_high_ subgroup had significant higher F_max_ (1087.42 ± 311.05 N, *p* = 0.01) and Mtk (4.71 ± 0.37 cm^2^, *p* = 0.03) values than the BMI_low_ (655.42 ± 114.76 N; 3.88 ± 1.96 cm^2^, respectively) (Table 2). However, there was no significant difference of F_max_ and Mtk between the BMI_high_ (782.44 ± 157.00 N, 5.12 ± 0.63 cm^2,^) and BMI_low_ (619.26 ± 68.50 N, 4.05 ± 0.51 cm^2,^) subgroups in the non-OC.

In the BMI_low_ group, the difference in the distribution of muscle fiber type 1 and 2 showed that type 2 was about 0.86% higher in the non-OC group. Contrastively, type 1 was about 18.46% higher in the OC group.

In the BMI_norm_ group, the difference in the distribution of muscle fiber type 1 and 2 showed that type 2 was about 4.16% higher in the non-OC group and 0.34% in the OC group.

In the BMI_high_ group, the difference in the distribution of muscle fiber types 1 and 2 showed that type 1 was about 18.76% higher in the non-OC group. Contrastively, type 2 was about 34.35% higher in the OC group. Among the BMI levels group, the difference in the distribution of muscle fiber type 1 and 2 showed the greatest difference in the BMI_high_ group (Table 3).

## 4. Discussion

The most important finding of the present study is that there were significantly greater values for F_max_ and Mtk in the BMI_high_ subgroup than the BMI_low_ subgroup among women consuming OC. The second most important finding of our study is that the OC-BMI_high_ group had the greatest difference in proportions between type 1 (32.96%) and type 2 (67.31%) fibers.

Previous studies have reported that younger individuals with obesity may have greater muscle strength than their non-obese counterparts [28,29]. Moreover, higher CSA values may be a result of the load imposed by a larger body weight acting as a chronic stimulus to the muscle tissue [43,44,45]. Maffiuletti et al. concluded that intramuscular fat associated with obesity may confound the relationship between Mtk and strength [45]. This finding may help explain the relatively high strength observed in overweight subjects. The present study shows similar results; participants with a BMI_high_ had greater F_max_ and Mtk values. However, the BMI_high_ subgroup in the non-OC group showed the lowest strength in terms of F_max_/Mtk, while the BMI_high_ subgroup in the OC group had the highest strength. More interestingly, although the non-OC group had thicker Mtks in all three subgroups, F_max_ and F_max_/Mtk were lower than all the subgroups in the OC group. Previous studies have reported a positive association between CSA and the percentage of type 2 muscle fibers in this population [46]. Concurrently, obese individuals have been shown to have a lower percentage of type 1 muscle fibers than normal-weight individuals [46]. The percentage of type 1 muscle fibers has been inversely related to body fat percentage [47].

Based on the results of previous studies, we analyzed muscle fiber composition to determine the proportion between type 1 and 2 muscle fibers according to BMI levels. In the OC group, a high BMI correlated with a high type 2 muscle fiber ratio, but in the non-OC group, the type 2 ratio did not increase with an increase in BMI. From these findings, it can be speculated that BMI and OC consumption may affect the levels of endogenous and exogenous estradiol and progesterone levels, as well as muscle strength, Mtk, and fiber composition. The impact of BMI on muscle composition and function may differ between OC and non-OC women due to the differences in substrate metabolism caused by endogenous and exogenous estradiol and progesterone during muscle strength training [4,48]. OCs alter steroid hormone concentrations; thus, exogenous and endogenous hormone level fluctuations may correspond to different types of muscle adaptations. Moreover, a high BMI in OC users may exert metabolic effects that could have an impact on physical performance [49].

However, some studies [13,50,51] reported no significant differences in muscle strength during menstrual cycle phases, suggesting that exogenous hormones alone may impact muscle strength. These findings are supported by Janse et al. [16]. Thus, most of the conflicting results in recent research on exercise performance on the menstrual cycle may be explained by methodological differences [16].

Many previous studies have reported that BMI may affect endogenous and exogenous hormone metabolism [52] because of the increased volume of distribution and altered plasma clearance in women with a high BMI. However, studies have also shown that different BMI levels impact strength training adaptation in women who consume OCs compared with those who do not. Thus, future research is required to determine the impact that different BMI levels have on muscle strength and structure in women who consume OCs compared with those who do not.

This study had several limitations. First, although nutrition plays an important role in muscle hypertrophy, we could not investigate its effect because the nutrient intake of the participants could not be assessed. Second, because muscle biopsy was only performed in participants who consented to it, certain analyses were performed on a smaller number of participants than others. Third, a previous study reported that it is necessary to measure an average of 150 muscle fibers to reduce variability [53]. In the present study, only five participants in the non-OC group and five participants in the OC group could be measured due to freeze damage in the muscle cells. Fourth, just leg-press strength was performed in this study; different results may have been obtained if a variety of muscle strengths had been included in the F_max_ measurement.

## 5. Conclusions

In this study, we found that there was a significant difference in F_max_ and Mtk according to the BMI level in the OC group, but no significant difference was found in the non-OC group. Moreover, the distribution of type 2 muscle fibers tended to be higher in the OC group of BMI_high_, although the sample size was small. Therefore, although no significant difference of F_max_ and Mtk was found according to BMI level in the non-OC group in this study, the increase in BMI level appeared to be more associative of muscle strength in the OC group. Based on the present results, future studies are needed that consider the BMI level as well as the presence or absence of OC in future research about women’s muscle strength.

## Figures and Tables

**Table 1:** Participants’ characteristics.

Characteristics	non-OC (*n* = 31)			OC (*n* = 27)		
	BMI_low_ (*n* = 10)	BMI_norm_ (*n* = 10)	BMI_high_ (*n* = 11)	BMI_low_ (*n* = 9)	BMI_norm_ (*n* = 9)	BMI_high_ (*n* = 9)
Age (y)	25.10 ± 4.61	26.18 ± 4.62	25.00 ± 4.69	24.25 ± 4.33	24.90 ± 4.09	25.20 ± 5.31
Height (m)	1.66 ± 0.04	1.62 ± 0.06	1.64 ± 0.04	1.65 ± 0.08	1.66 ± 0.06	1.67 ± 0.06
Weight (kg)	49.70 ± 3.59	58.91 ± 5.03	75.00 ± 5.13	48.13 ± 6.13	60.80 ± 4.32	77.20 ± 4.85
BMI (kg/m^2^)	17.94 ± 1.14	22.43 ± 1.19	27.84 ± 1.91	17.67 ± 0.95	22.13 ± 0.99	27.28 ± 1.65

non-OC, not taking oral contraceptive; OC, taking oral contraceptive; BMI_low_, body mass index < 18.5; BMI_norm_, 18.5 ≤ body mass index < 25; BMI_high_, body mass index ≥ 25. Values are presented as mean ± standard deviation.

**Table 2:** F_max_, Mtk, and F_max_/Mtk for the BMI_low_, BMI_norm_, and BMI_high_ subgroups in the non-OC and OC.

Variables	non-OC (*n* = 31)					OC (*n* = 27)				
	BMI_low_ (*n* = 10)	BMI_norm_ (*n* = 10)	BMI_high_ (*n* = 11)	*F*	*p*	BMI_low_ (*n* = 9)	BMI_norm_ (*n* = 9)	BMI_high_ (*n* = 9)	*F*	*p*
F_max_ (N)	619.26 ± 68.50	657.09 ± 128.18	782.44 ± 157.00	1.65	0.23	655.42 ± 114.76	842.02 ± 136.32	1087.42 ± 311.05	5.77	0.01 *
Mtk (cm^2^)	4.05 ± 0.51	4.34 ± 0.75	5.12 ± 0.63	2.19	0.15	3.88 ± 1.96	4.15 ± 0.37	4.71 ± 0.37	4.52	0.03 *
F_max_/Mtk (N)	155.62 ± 31.36	156.31 ± 44.79	152.00 ± 18.72	0.14	0.99	168.66 ± 36.26	203.06 ± 27.93	232.46 ± 71.54	1.94	0.18

OC, taking oral contraceptive; non-OC, not taking oral contraceptive; F_max_, maximum isometric force; Mtk, sum of m. rectus femoris and m. vastus lateralis muscle thickness; F_max_/Mtk, F_max_ divided by Mtk; BMI_low_, body mass index < 18.5; BMI_norm_, 18.5 ≤ body mass index < 25; BMI_high_, body mass index ≥ 25. Values are presented as the mean ± standard deviation. * Significantly different compared to the BMI_low_ and BMI_high_ (*p* < 0.05).

**Table 3:** Proportion of muscle fiber types 1 and 2 from the vastus lateralis for the BMI_low_, BMI_norm_, and BMI_high_ subgroups in the non-OC and OC.

Group	non-OC (*n* = 5)		OC (*n* = 5)	
Muscle fiber type	Type 1	Type 2	Type 1	Type 2
BMI_low_ (%)	49.57	50.43	59.23	40.77
BMI_norm_(%)	47.92	52.08	49.83	50.17
BMI_high_(%)	59.38	40.62	32.96	67.31

OC, taking oral contraceptive; non-OC, not taking oral contraceptive; BMI_low_, body mass index < 18.5; BMI_norm_, 18.5 ≤ body mass index < 25; BMI_high_, body mass index ≥ 25.

## Data Availability

Data sharing is not applicable to this article.
